# Research Progress in Prognostic Factors and Biomarkers of Ovarian Cancer

**DOI:** 10.7150/jca.47695

**Published:** 2021-05-13

**Authors:** Shuna Liu, Ming Wu, Fang Wang

**Affiliations:** 1 Department of Laboratory Medicine, the First Affiliated Hospital of Nanjing Medical University, Nanjing, China, 210029.; 2 National Key Clinical Department of Laboratory Medicine, Nanjing, China, 210029.

**Keywords:** ovarian cancer, prognostic factor, biomarker

## Abstract

Ovarian cancer is a serious threat to women's health; its early diagnosis rate is low and prone to metastasis and recurrence. The current conventional treatment for ovarian cancer is a combination of platinum and paclitaxel chemotherapy based on surgery. The recurrence and progression of ovarian cancer with poor prognosis is a major challenge in treatment. With rapid advances in technology, understanding of the molecular pathways involved in ovarian cancer recurrence and progression has increased, biomarker-guided treatment options can greatly improve the prognosis of patients. This review systematically discusses and summarizes existing and new information on prognostic factors and biomarkers of ovarian cancer, which is expected to improve the clinical management of patients and lead to effective personalized treatment.

## Introduction

Ovarian cancer is the most fatal gynecological tumor, its incidence is next to cervical cancer and endometrial cancer, but its mortality rate is the first among reproductive system malignancies. According to the data of cancer statistics in 2020, the number of new cases is about 21750 and the number of deaths is 13940 1. Ovarian is located in the posterolateral uterine bottom, the onset is insidious, the early symptoms lack specificity, and the screening effect is limited, so the early diagnosis of ovarian cancer is difficult. According to the American congress of obstetricians and gynecologists (ACOG), 70 to 75 percent of ovarian cancers are diagnosed late, and the 5-year survival rate for most women is 20 to 30 percent 2. Compared with other gynecological tumors, ovarian cancer has complex pathological types, high recurrence rate and poor prognosis. Patients with distant metastasis due to delayed medical treatment and tolerance to chemotherapy have worse prognosis. Therefore, the identification of effective clinical prognostic factors and biomarkers is crucial to improve the prognosis of ovarian cancer patients. With the in-depth study of the molecular changes that drive the transformation of ovarian cancer and tumor progression, many new molecular analysis techniques have been widely used. Recent studies have shown that microRNAs (miRNAs) may play an important role in the pathogenesis of ovarian cancer and serve as potential biomarkers 3.

The main contents of this review are divided into two parts: classic prognostic factors and novel prognostic factors. Classic prognostic factors included clinicopathologic factors (FIGO stage, degree of differentiation, degree of tumor reduction surgery, course of chemotherapy) and serum CA125. New prognostic factors mainly include blood- or tissue-based biomarkers. The ovarian cancer field has lagged in incorporating targeted therapies into standard treatments, these novel biomarkers are expected to provide therapeutic targets for ovarian cancer, thus guiding clinical practice, improving patient prognosis and ultimately reducing the risk of death of ovarian cancer patients.

## Search Methods

Based on the topics discussed in this review, we systematically searched the recent medical literatures on novel prognostic biomarkers of ovarian cancer in PubMed and PMC databases by using our search strategy. All the literatures included in the study were published between February 1, 2015 and February 1, 2021. After excluding the duplicated literatures in the two databases, a total of 1,923 literatures met the restriction conditions. Then the retrieved literatures were imported into the literature management software Endnote. Preliminary screening was performed by reading the titles and abstracts of the literatures to exclude irrelevant studies, and then the full text of the included literatures was evaluated. In order to ensure the reliability of the research results, we only selected studies with more than 50 ovarian cancer patients, and the biomarkers studied in the literature were consistent with the clinical results. The inclusion and exclusion criteria and search strategy are provided in the appendix. Finally, a manual search was conducted in major journals and the reference lists of the selected papers to find other relevant citations that were missing by the electronic search.

## Search Results

**Figure 1:**
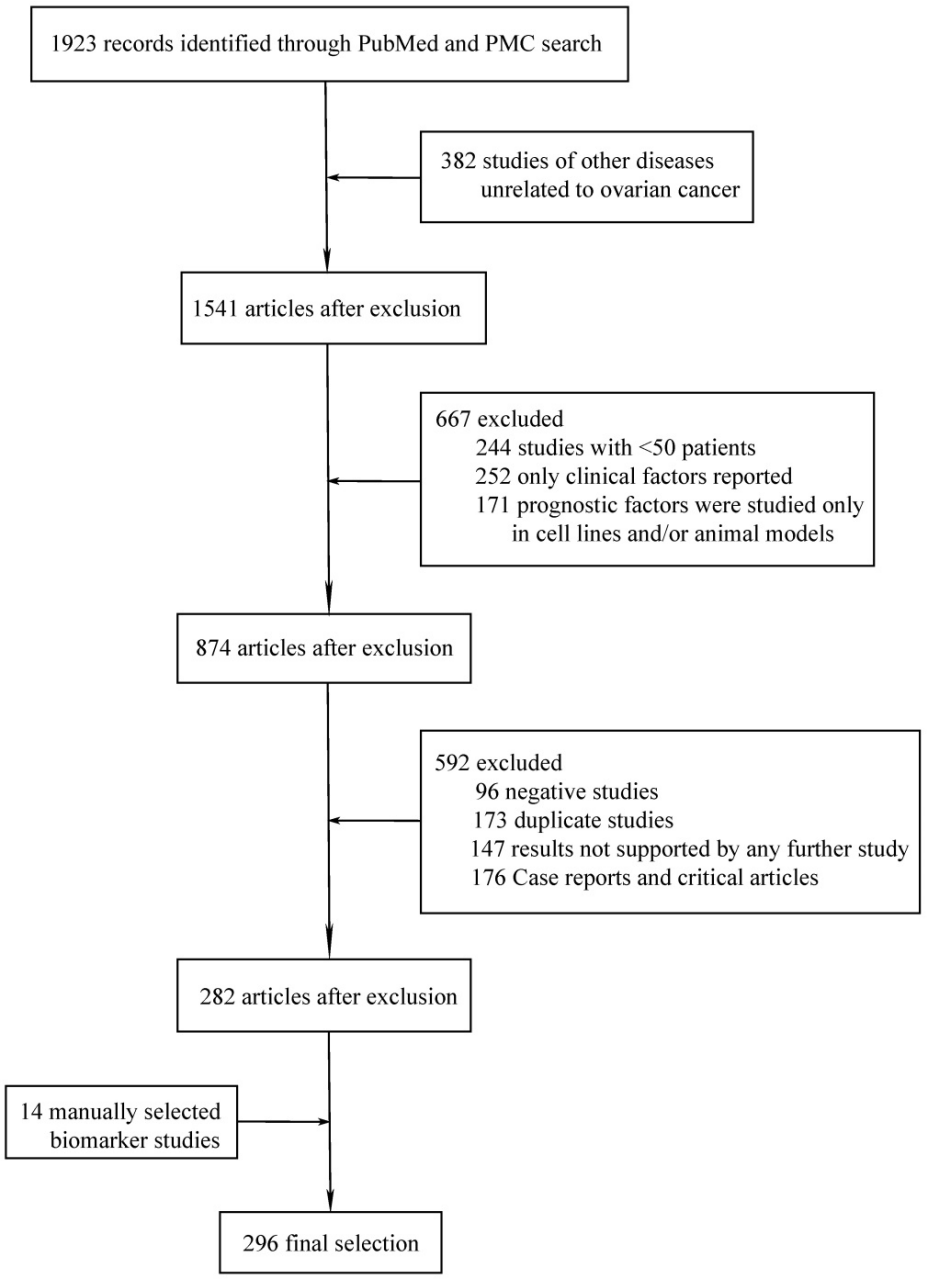
Flowchart of article selection process.

A total of 297 different novel prognostic biomarkers were reported in 296 studies that met the inclusion criteria (Figure 1). These prognostic biomarkers were classified according to the purpose of the study; there were 45 studies on biomarkers in the blood of ovarian cancer patients (Table 1) and 251 studies on biomarkers in tumor tissues (Tables 2-4).

## Classic prognostic factors

Clinicopathologic factors and serum CA125 level are independent factors affecting the prognosis of ovarian cancer patients, which have been widely used to guide accurate and reasonable clinical treatment, so as to improve the survival rate of patients.

### Clinicopathological factors

The clinicopathological factors that affect the prognosis of ovarian cancer mainly include: FIGO stage, degree of differentiation, degree of tumor reduction surgery, course of chemotherapy. Previous literature has reported the importance of ovarian cancer staging for prognosis and treatment options, ovarian cancer can be classified as stage I-IV according to FIGO staging criteria, and most patients have stage III disease. Studies have shown that patients with stage I ovarian cancer have a 5-year survival rate of more than 90%; when ovarian cancer is confined to the pelvis (stage II), the estimated 5-year survival rate is about 70%; when ovarian cancer has spread to the entire abdominal cavity (stage III) or to distant parts (stage IV), the 5-year survival rate is less than 30% 4. The survival prognosis of patients in the early stage was significantly better than that in the late stage. Differentiation degree of ovarian cancer includes high differentiation, moderate differentiation and low differentiation (poor differentiation), there has been evidence that poor differentiation of ovarian cancer is associated with worse survival. A large sample study established a predictive model for overall survival in 1189 patients with primary ovarian epithelial carcinoma, cox regression analysis showed that the worse the differentiation, the greater the risk of death 5.

Surgery is the most effective treatment for ovarian cancer, once suspected for ovarian cancer, should be performed as early as possible. Staging surgery is performed for early stage cancer, including resection of the tumor and definite staging. Tumor cell reduction was performed for advanced cancer, and the primary tumor and all metastases were removed as far as possible to minimize the number of tumor cells. Studies have confirmed that the degree of tumor cell reduction and the number of residual lesions after the first operation are important prognostic factors for advanced ovarian cancer 6. The research of Jing shui et al. shows that the size of residual tumor foci was negatively correlated with the survival rate of patients and those with residual tumor foci ≤ 2 cm had better prognosis 7. It is helpful to improve the prognosis and long-term survival rate of patients by minimizing or removing residual tumor foci.

Chemotherapy is an important adjuvant treatment for ovarian cancer, and most ovarian cancer is sensitive to chemotherapy. Platinum-based drugs (cisplatin and carboplatin) and taxanes (paclitaxel and docetaxel) are chemotherapy drugs commonly used in the treatment of ovarian cancer 8. Postoperative adjuvant chemotherapy should follow the principles of standard, early and adequate course of treatment. Currently, it is generally considered that the standard course of chemotherapy for ovarian cancer is 6 courses. Three trials of primary advanced ovarian cancer compared the efficacy of chemotherapy with cisplatin in 5-6 cycles and 8-12 cycles, and the results showed that there was no benefit after 6 cycles of chemotherapy 9. Another study on prognostic factor analysis of 129 cases of epithelial ovarian cancer showed that the median OS of patients with postoperative chemotherapy course ≥ 6 courses was significantly higher than that of patients with less than 6 courses of chemotherapy, and the difference was statistically significant (*P*<0.0001). There was no statistically significant difference in median OS in patients with 6 courses of chemotherapy, 7 courses of chemotherapy, 8 courses of chemotherapy or more than 8 courses of chemotherapy (*P*=0.816) 10. In summary, postoperative chemotherapy course is an important prognostic factor for ovarian cancer, and standard chemotherapy course is associated with higher overall survival.

### CA125

CA125, encoded by the MUC16 gene, is a classic marker for the diagnosis of ovarian cancer and was first described in the study of Bast RC et al 11. Serum CA125 lacks sensitivity and specificity and cannot be used as a single marker for early detection of ovarian cancer 12,13, but the CA125 value after surgery and chemotherapy plays an important role in monitoring recurrence and evaluating prognosis. Redman et al. detected the CA125 value before the third chemotherapy in 78 patients with stage II~IV ovarian cancer after the completion of two courses of chemotherapy, and the analysis showed that those with CA125 ≤ 35U/mL had a 1-year survival rate of 96%, while those with CA125>35U/mL had a 1-year survival rate of 15% 14. The half-life of CA125 is another widely reported indicator. In some studies, CA125 was regularly detected after surgery and chemotherapy in 225 patients with advanced ovarian cancer, and the complete remission rate of patients with serum CA125 half-life <25 d was found to be 3.6 times higher than that of patients with >25 d through analysis combined with the results of secondary exploration 15. Therefore, continuous monitoring of CA125 is of great value for efficacy evaluation and prognosis analysis of ovarian cancer patients.

## Novel prognostic factors

In order to develop a powerful predictive tool with both sensitivity and specificity to monitor ovarian cancer response to treatment, the research on prognostic biomarkers for ovarian cancer is continuously advancing.

### Blood-based prognostic biomarkers

Blood test is minimally invasive, simple and easy to obtain specimens, and blood test results are widely used in clinic to assist the guidance of treatment. A variety of novel prognostic biomarkers derived from blood can provide a new tool for the clinical management of ovarian cancer. A total of 43 blood based biomarker studies met our selection criteria (Table 1), of which 13 were evaluated using ELISA methods for protein biomarkers 16,18-22,30-32,34,36,37,44. PCR technology was used for detection of DNA or RNA source biomarkers 17,33,40-42,46,47,49,53,54,57. The 41 novel prognostic biomarkers provided by 43 studies can be classified by biological function, including cell proliferation and invasion 16-22, inflammatory response 23-29, angiogenesis 30-33, antioxidant 34, immune response 35-39, chemotherapeutic sensitivity 40-44, mitosis process 45, EMT (epithelial-to-mesenchymal transformation) and metastasis 46,47, deregulation of the cellular transport 48 and apoptosis process 31. The following are representative novel prognostic factors reported in the literature.

A large number of studies have shown that chronic inflammation is closely related to the occurrence and development of cancer, and a variety of inflammatory cells and inflammatory factors participate in and promote the proliferation, invasion and metastasis of tumor cells, and affect the prognosis of patients 310. Neutrophils and lymphocytes are both important cells involved in the inflammatory response process. The changes in the number of them can directly reflect the degree of inflammatory response in the body. NLR (neutrophil to lymphocyte ratio) is an important biological indicator of systemic inflammatory response, which can be obtained by calculating the ratio after the complete blood count 311. Previous studies have shown that elevated NLR is an independent prognostic risk factor for several malignant tumors, including ovarian cancer 312-314. The study of Stanislaus Argeny et al. found that the non-specific inflammatory response in cancerous tissues would lead to changes in the level of peripheral blood cells, mainly manifested as an increase in NLR. Studies have shown that neutrophils can alter the tumor microenvironment by producing cytokines and chemokines, they also promote the transformation of normal cells into tumor cells by secreting substances like reactive oxygen species and proteases. Moreover, the migration and diffusion ability of tumor cells can be enhanced by secreting platelet activating factor, matrix metalloproteinase and other factors related to tumor cell metastasis. In addition, lymphocytes are important components of the immune system and play an important role in immune surveillance. The decreased number of lymphocytes indicates the weakened immunity of the body and the reduced monitoring and killing effect on tumor cells, which cannot effectively prevent the proliferation and migration of tumor cells. Therefore, an elevated preoperative NLR usually indicates a poor prognosis in ovarian cancer patients 315. The study of Zhang H et al. suggested that NLR could be used to differentiate CA125-negative ovarian cancer and was superior to CA125 in predicting patients' overall survival (OS) and progression free survival (PFS) 316. In addition, a multivariate analysis of clinical data in 165 initial treatment ovarian cancer patients also suggested that NLR is an independent prognostic factor for PFS and OS in ovarian cancer patients 28.

Alterations in energy metabolism are a decisive biochemical feature of tumor cells, in other words, abnormal activation of glycolytic pathway still exists in tumor cells even under the condition of sufficient oxygen supply, consume large amounts of glucose and eventually produce lactic acid in order to satisfy energy supply of malignant tumor cell proliferation, this phenomenon is called aerobic glycolysis of tumors, also known as the Warburg effect 317. In the process of glycolysis of malignant tumors, there is an important catalytic enzyme, namely lactate dehydrogenase (LDH), which mainly catalyzes the exchange of pyruvate and lactic acid, and is highly expressed in hypoxic cells, especially in tumor cells. Compared with normal tissues, the levels of glycolysis in malignant tissues were higher, and the serum LDH level of patients increased with the progression of the disease, especially in the advanced stage of the tumor 318. A study shows that the LDH levels at different stages and grades differed significantly in ovarian cancer, survival curves revealed that higher LDH expression was correlated with shorter survival (*P*<0.05). In addition, SATB1 may reprogram energy metabolism in ovarian cancer by regulating LDH and MCT1 levels to promote metastasis 319. As another marker of tissue damage and inflammation, elevated serum LDH level can promote the proliferation, metastasis and development of cancer cells, which is commonly seen in a variety of malignant tumors 320,321. A study showed that preoperative higher LDH levels were significantly associated with poor survival in patients with high grade serous ovarian cancer through survival analysis, serum high LDH levels are a promising prognostic biomarker 26.

Mesothelial protein (MSLN) is a cell surface glycoprotein, which was found by Chang et al. 322 and is usually only expressed in mesothelial tissue of body cavity. In recent years, MSLN as a differentiation antigen has been proved to be overexpressed in malignant pleural mesothelioma, pancreatic cancer, ovarian cancer and other malignant tumors, and may through increased synthesis of cyclinD1 and suppress the degradation and forming MSLN/MUC16 complex pathways involved in tumor cell proliferation, adhesion and transfer process, it is related to transcoelomic spread of ovarian cancer cells 323. In addition, MSLN inhibits paclitaxel-induced apoptosis through serine and threonine kinase pathways, leading to chemotherapy resistance and seriously affecting the prognosis of patients 324. The study of Karolina Okla et al. confirmed that plasma MSLN concentration in EOC patients was significantly higher than that in benign ovarian tumor patients and healthy women. Kaplan-Meier analysis results showed that, compared with low MSLN level, only high MSLN concentration of EOC patients before treatment was significantly correlated with a shorter 5-year OS (*P*=0.03), which predicted poor prognosis 21. Another study showed that MSLN can enhance the invasion of ovarian cancer by inducing MMP-7 through MAPK/ERK and JNK pathways, blocking the MSLN-related pathway may be a potential strategy to improve the prognosis of ovarian cancer patients 325.

Aurora A kinase (AAK) is encoded by the Aurka gene and is a member of the serine/threonine kinase family. And as an important mitotic regulator, it can participate in many processes of cell mitosis and maintain chromosome division and spindle stability together with centrosomes 326. Overexpression of Aurora A has been observed in a variety of malignant tumor types and plays an important regulatory role in the key control points of the tumorigenic transformation response through p53/TP53 phosphorylation 327. Aurora A overexpression can also lead to abnormal amplification of centrosomes, leading to multilevel allocation and instability of chromosomes during division, and then to activation of oncogenes or inactivation of tumor suppressor genes 328. Through gene chip screening and RT-PCR, the study of Hellleman et al. confirmed that Aurora A was overexpressed in ovarian cancer tissues that did not respond to platinum therapy, compared with ovarian cancer patients who responded to platinum therapy, and patients with overexpression of Aurora A had a poor prognosis 329. A single nucleotide polymorphism in G169A at codon 57 of Aurora A locus leads to the substitution of valine by isoleucine, leading to the production of variant II. Kimura et al. 45 showed that AAK activity was reduced by the II variant, and the inhibited AAK could lead to cell death by affecting the mitosis process. Therefore, the change of single nucleotide polymorphisms in AAK may be a protective factor for cancer risk.

Galectin is an important member of the lectin superfamily, it is widely expressed in a variety of cell types and plays an important role in apoptosis, angiogenesis, cell migration, and tumor immune escape. Dysfunction or altered expression of galectin is associated with a variety of cancer types 330. Galectin-8 and galectin-9 both have two carbohydrate recognition domains and are tandem repeat galactosins that regulate a variety of biological functions, including cell aggregation, cell adhesion, and tumor cell apoptosis 331. Recent studies have shown that galectin-9 promotes CD8 ^+^ T cell failure and induces proliferation of myeloid inhibitory cells by binding to T cell immunoglobulin mucin 3 (Tim-3), thereby participating in immune escape of tumor cells 332. In addition, the expression of galectin-8 in solid tumors has also been proved to be closely related to tumor cell adhesion or metastasis 333. Labrie M et al. showed that plasma Gal-8 and Gal-9 levels were significantly increased in HGSOC patients compared to healthy controls, and higher plasma galectin-8 and galectin-9 levels were associated with a shorter 5-year disease-free survival (DFS) and 5-year OS (*P*=0.005), multivariate analysis further demonstrated that both plasma galectin-8 and galectin-9 could be promising biomarkers for poor prognosis in high grade serous ovarian cancer patients 171.

Angiogenesis plays an important role in tumor growth and metastasis. Neovascularization provides oxygen and nutrients to tumor cells, which can enhance cell proliferation and invasion ability 334. Tumor tissue can secrete a variety of proangiogenic substances to induce and regulate angiogenesis, among which vascular endothelial growth factor (VEGF) is the primary stimulator of tumor angiogenesis. VEGF family members include VEGF-A, VEGF-B, VEGF-C, VEGF-D, etc. Among them, the biologic activity of VEGF-A is the most important, which can promote neovascularization and increase vascular permeability through VEGF/VEGFR (Vascular Endothelial Growth Factor Receptor) signaling pathway 335. Previous studies have shown that VEGF-A is closely related to the occurrence and development of cancer and some inflammatory diseases 336. Studies have investigated the efficacy of serum VEGF-A levels as prognostic markers in Epithelial ovarian cancer (EOC) patients, the experiment confirmed that the OS of patients with high VEGF-A level was significantly lower than that of patients with low VEGF-A level, and the difference was statistically significant (*P*=0.015). Moreover, the VEGF-A level of patients was correlated with FIGO stage. Multivariate analysis showed that serum VEGF-A could be an independent prognostic factor for OS of patients 32. The study of Dobrzycka B et al. showed that serum VEGF level was significantly increased in patients with serous ovarian cancer (SOC) compared with healthy control group, and higher serum VEGF level was significantly correlated with poor prognosis, and multivariate analysis confirmed that serum VEGF level was an independent risk factor for prognosis 31.

MicroRNAs (miRNAs) are a class of single-stranded small RNAs encoded by endogenous genes, which regulate the expression of target genes by acting on target mRNA to promote its degradation or inhibit its translation 337. MiRNAs are involved in the regulation of a variety of human life activities, and studies have found that miRNAs are closely related to the occurrence and development of a variety of malignant tumors 338,339. At present, more than 50% miRNA genes have been located in tumor-related chromosomal rearrangement regions, which have important research and application values in the diagnosis, treatment and prognosis prediction of malignant tumors. EMT is closely related to tumor invasion and metastasis, many miRNAs have been proved to directly regulate the expression of epithelial markers and indirectly regulate EMT-related growth factor signaling pathways and transcription factors to affect the EMT process 340,341. At present, miR-200 family is the most studied miRNA related to EMT process. Gregory et al. found that TGF- Beta/ZEB/miR-200 signaling pathway can regulate the transformation of cell epithelial-mesenchymal phenotype 342. MiR-200c and miR-141 belong to the microRNA-200 family, Gao,Y.C. et al. evaluated the value of these two miRNAs as novel prognostic biomarkers for ovarian cancer. Studies have shown that the expression levels of serum miR-200c and miR-141 in ovarian cancer patients are significantly increased compared with the normal control group, and the expression levels of the two miRNAs are correlated with different stages and pathological subtypes of ovarian cancer. Survival analysis showed that compared with the group with high serum miR-200c expression, the overall survival rate of the group with low serum miR-200c expression was significantly reduced. This is similar to the analysis results of different miR-141 expression groups, so both miR-200c and miR-141 are likely to be promising prognostic biomarkers for ovarian cancer 49. Another study compared the expression levels of miR-200a, miR-200b and miR-200c in blood samples from 70 EOC patients and healthy controls, the results showed that these three miRNAs were significantly higher expressed in serum samples from EOC patients compared to normal controls, statistical analysis confirmed that the high expression of miR-200a, miR200b and miR-200c was significantly correlated with tumor histological subtypes, stages and lymph node metastasis, and all of them could be used as reliable indicators for predicting the prognosis of patients with EOC 46.

### Tissue-based prognostic biomarkers

The overwhelming majority of selected biomarker studies investigated different tissue-based biomarkers using a variety of technical research methods. The selected tissue prognostic biomarkers can be divided into immunohistochemical biomarkers (68.77%) 59-232, DNA biomarkers (3.95%) 159,233-241 and RNA biomarkers (27.28%) 242-309. The prognostic value of 172 protein biomarkers was evaluated by immunohistochemistry in 174 studies (Table 2). These markers are classified according to their biological functions, mainly including such functional pathways as EMT and metastasis 59-71, inflammation and immunity 72-84, antioxidant 85,86, angiogenesis 87-99, cell proliferation, migration and invasion 100-116, chemotherapeutic sensitivity 117-197 and cell cycle regulation 198-201. The remaining 79 studies of prognostic biomarkers were based on genomic DNA or RNA (Tables 3-4), involving different functional pathways in the progression of ovarian cancer, such as gene locus methylation 159,233-235, mutation status 237,238, gene polymorphism 240,241 and the expression of non-coding RNA during cancer cell proliferation, migration and invasion 242-282.

As a new type of anti-tumor effector lymphocytes with potential therapeutic value, the correlation between TIL and patient prognosis and survival has been widely concerned. Through systematic literature retrieval, we determined that TIL is a promising prognostic biomarker, and its level can be detected by immunohistochemistry. TIL can be classified by function and location in the tumor tissue, which is generally associated with better prognosis and survival, in which the presence of CD8^+^ T cells is positively correlated with survival 343,344. The presence of TIL in a variety of tumor types, including metastatic melanoma, breast cancer, colorectal cancer, and ovarian cancer, has been found to be significantly correlated with patient clinical outcomes and is an important positive prognostic factor 345-349. There is evidence that ovarian cancer patients are usually accompanied by systemic immunosuppression. In contrast, patients with a stronger immune response have improved survival and respond better to chemotherapy 350. Mauricio P et al. 81 evaluated TIL as a prognostic survival indicator for a group of HGSOC patients, and examined the expression of matrix and intraepithelial TIL (CD4^+^ and CD8^+^) in tissue samples. Multivariate analysis showed that intraepithelial CD4^+^ TIL infiltration was associated with better PFS and OS, intraepithelial CD8^+^ TIL infiltration was only associated with better PFS. This confirms previous studies that ovarian cancer patients with high infiltration of CD4^+^ and CD8^+^ TIL have better prognosis. As a new method for the treatment of ovarian cancer, the potential value of targeted immunotherapy is an important research direction, which can be used to guide clinical practice, reduce recurrence and improve the long-term survival rate of patients.

Mitochondrial superoxide dismutase (MnSOD or SOD2) is the most important antioxidant enzyme in mitochondria, which protects cells from oxidative damage induced by reactive oxygen species (ROS) and lipid peroxidation by converting endogenous superoxide to hydrogen peroxide 351. Studies have demonstrated that SOD2 overexpression can enhance the invasion and metastasis of tumor cells by increasing the expression of matrix metalloproteinases (MMP) family members or activating Redox sensitive signaling pathways 352. New evidence suggests that inhibition of SOD2 activity in tumor cells leads to increased apoptosis, inhibition of proliferation and increased sensitivity to chemotherapeutics 353. There is growing evidence that SOD2 overexpression is associated with poor prognosis in a variety of cancer types, including renal clear cell carcinoma and ovarian cancer 354-356. A study based on SOD2 immunohistochemical staining confirmed the correlation between SOD2 expression and patient prognosis in the endometriosis-associated ovarian cancer (EAOC) case group. Kaplan-Meier analysis showed that high SOD2 expression was associated with shorter PFS (*P*=0.0669) and poorer OS (*P*=0.0405), and increased SOD2 expression was a predictive biomarker for poor prognosis in EAOC 86.

Genome-wide analysis has confirmed that epigenetic changes are common events in many cancers, cellular genomic epigenetic disorders are important causes of many diseases, including cancer and autoimmune diseases. Epigenetic changes in human malignancies mainly include DNA methylation, nucleosomal remodeling histone modification and non-coding RNA dysregulation 357. Numerous studies have confirmed that abnormal methylation of multiple genes involved in DNA repair, Akt /mTOR, Redox response, apoptosis, cell adhesion and cancer stem cell signaling pathways are associated with poor prognosis in ovarian cancer patients 358. Mase et al. 235 confirmed that the DNA methylation status of ZNF671 was closely related to the recurrence and prognosis of patients with serous ovarian cancer. Multiple analysis methods combined showed that the methylation status of ZNF671 was an independent factor to predict the early recurrence of patients and patients with DNA methylation of ZNF671 had poor prognosis (*P*<0.05). A subsequent study validated the prognostic significance of HS3ST2 methylation in patients with advanced EOC in three separate dataset of TSGH, AOCS, and TCGA, studies have confirmed that HS3ST2 inhibits the malignant phenotype of ovarian cancer by interfering with various carcinogenic ligand signals, such as IL-6, FGF2 and EGF, and patients with low HS3ST2 expression accompanied by high expression of carcinogenic cytokines or growth factors have the worst prognosis 159. In conclusion, abnormal DNA methylation in tumor cells can be used as an effective prognostic marker for ovarian cancer. Non-coding RNA is an important part of epigenetic changes, among which long non-coding RNA (lncRNA) is an emerging regulatory RNA that is involved in the regulation of a variety of physiological and pathological processes and is abnormally expressed in a variety of types of cancers. It has been reported that the differential expression of lncRNA in ovarian cancer, lung cancer, gastric cancer and liver cancer is related to the prognosis of patients 359. Cao Y et al. 265 confirmed that the expression of lncRNA CCAT1 was up-regulated in EOC tissues, and the high expression of lncRNA CCAT1 could promote the process of EMT of EOC cells, and enhance the migration and invasion ability of cells. Furthermore, high lncRNA CCAT1 expression was associated with FIGO stage, histological grade, lymph node metastasis and poor survival. Multivariate cox regression analysis showed that CCAT1 expression was an independent prognostic factor. In addition, it has been demonstrated that silencing of lncRNA CCAT2 in cancer cells significantly inhibits cell proliferation, migration and invasion through the Wnt/β-catenin signaling pathway, and the results of subsequent survival analysis showed that high CCAT2 expression was associated with shorter OS or DFS, cox proportional risk regression model analysis showed that CCAT2 expression level was an independent prognostic indicator for overall survival, and these data results confirmed that lncRNA CCAT2 was a reliable prognostic marker for ovarian cancer 269.

## Conclusion

Ovarian cancer is the most fatal gynecological malignancy with high incidence and low survival rate. By exploring the prognostic biomarkers associated with ovarian cancer recurrence and progression, independent risk factors affecting patient prognosis were identified, which laid a solid foundation for the development of novel treatment strategies and the improvement of patient treatment outcomes. This review searched the literature and database for the relevant reports on prognostic biomarkers of ovarian cancer, reviewed the classic clinical prognostic biomarkers, and focused on the recently discovered various prognostic markers. Advances in genomics, proteomics and metabolomics have provided favorable conditions for the discovery of novel prognostic biomarkers that have identified a variety of promising prognostic biomarkers, including miRNA, lncRNA and TIL, these biomarkers can affect the prognosis of patients through a variety of biological functional pathways. TCGA data sets and public databases can provide data information for large patient cohort genome studies, the application of bioinformatics modeling and high-throughput molecular analysis techniques has greatly enriched the knowledge related to biological processes such as cancer progression. The prognostic value of a variety of novel biomarkers was evaluated by integrating genomic, proteomic and metabolomic data and clinical information with a multivariate analysis model. The effectiveness of these novel prognostic biomarkers still needs to be further validated in large clinical trials. By studying the functional pathways of regulation of these molecular markers, the potential molecular mechanisms are revealed, so as to identify new therapeutic targets. This is a high-precision medical method, which may promote personalized treatment of ovarian cancer patients and improve their prognosis.

## Supplementary Material

Supplementary materials.

## Figures and Tables

**Table 1:** Blood-based biomarkers in ovarian cancer

	Expression or ratio	Potential clinical use	Example study			
			Study	Studied biomarkers	Subsite	Patients(n)
**Cell proliferation and invasion**						
Leptin	Increased	Poor prognosis	Kato, S., et al. (2015)^16^	Leptin	EOC	70
miR-429	Increased	Good prognosis	Meng, X., et al. (2015)^17^	miR-429	EOC	180
ADAM12	Increased	Poor prognosis	Cheon, D. J., et al. (2015)^18^	ADAM12	HGSOC	84
Septin-9, clusterin	Increased	Poor prognosis	Lyu, N., et al. (2018)^19^	Septin-9, clusterin	EOC	137
MMP3, TIMP3	Increased	Poor prognosis	Cymbaluk-Ploska, A., et al. (2018)^20^	MMP3, TIMP3	OC	104
MSLN	Increased	Poor prognosis	Karolina Okla et al. (2018)^21^	MSLN	EOC	97
CYFRA21-1	Increased	Poor prognosis	Jin, C., et al. (2019)^22^	CYFRA21-1	EOC	203
**Inflammation**						
NLR	Increased	Poor prognosis	Feng, Z., et al. (2016)^23^	NLR	HGSOC	875
NLR	Increased	Poor prognosis	Li, Z., et al. (2017)^24^	NLR	EOC	654
CRP / Alb	Increased	Poor prognosis	Liu, Y., et al. (2017)^25^	CRP/Alb	OC	200
NLR, LDH	Increased	Poor prognosis	Mauricio, P., et al. (2018)^26^	NLR, LDH	HGSOC	128
AFR	Decreased	Poor prognosis	Yu, W., et al. (2019)^27^	AFR	EOC	313
NLR	Increased	Poor prognosis	Ceran, M. U., et al. (2019)^28^	NLR	EOC	244
PLR	Increased	Poor prognosis	Ceran, M. U., et al. (2019)^28^	PLR	EOC	244
NLR	Increased	Poor prognosis	Nomelini, R. S., et al. (2019)^29^	NLR	OC	72
**Angiogenesis**						
Fibulin-4	Increased	Good prognosis	Chen, J., et al. (2015)^30^	Fibulin-4	OC	160
VEGF	Increased	Poor prognosis	Dobrzycka, B., et al. (2015)^31^	VEGF	SOC	92
VEGF-A	Increased	Good prognosis	Komatsu, H., et al. (2017)^32^	VEGF-A	EOC	128
LncRNA MALAT1	Increased	Poor prognosis	Qiu, J. J., et al. (2018)^33^	LncRNA MALAT1	EOC	60
**Antioxidant**						
8-OHdG	Increased	Poor prognosis	Pylväs-Eerola, M., et al. (2015)^34^	8-OHdG	EOC	112
**Immune response**						
TNFa/IL-4 ratio	Increased	Good prognosis	Hao, C. J., et al. (2016)^35^	TNFa/IL-4 ratio	OC	50
sPD-L1	Increased	Poor prognosis	Chatterjee, J., et al. (2017)^36^	sPD-L1	EOC	71
s-CD95L	Increased	Good prognosis	De La Motte Rouge, T., et al. (2019)^37^	s-CD95L	HGSOC	51
absolute lymphocyte count	Decreased	Poor prognosis	Lee, Y. J., et al. (2019)^38^	absolute lymphocyte count	OC	537
CD4/CD8 ratio	Decreased	Good prognosis	Waki, K., et al. (2020)^39^	CD4/CD8 ratio	OC	52
**Chemotherapeutic sensitivity**						
CEBPA, C.69.OG>T polymorphism (rs34529039)	Increased	Poor prognosis	Konopka, B., et al. (2016)^40^	CEBPA, C.69.OG>T polymorphism (rs34529039)	OC	118
hyperfibrinogenemia	Increased	Poor prognosis	Feng, Z., et al. (2016)^41^	hyperfibrinogenemia	HGSOC	875
ERCC1	Expression	Poor prognosis	Chebouti, I., et al. (2017)^42^	ERCC1	OC	65
miR-135a-3p	Increased	Good prognosis	Fukagawa, S., et al. (2017)^43^	miR-135a-3p	OC	98
Gal-8, Gal-9	Increased	Poor prognosis	Labrie, M., et al. (2017)^44^	Gal-8, Gal-9	HGSOC	160
**Mitotic process**						
Aurora A codon 57 SNP	Increased	Good prognosis	Niu, H., et al. (2017)^45^	Aurora A codon 57 SNP	OC	122
**EMT and metastasis**						
miR 200a, miR 200b, miR 200c	Increased	Poor prognosis	Zuberi, M., et al. (2015)^46^	miR 200a, miR 200b, miR 200c	EOC	70
miR-200b, miR-200c	Increased	Poor prognosis	Meng, X., et al. (2016)^47^	miR-200b, miR-200c	EOC	163
**Deregulation of the cellular transport**						
KPNA2	Increased	Poor prognosis	Huang, L., et al. (2017)^48^	KPNA2	EOC	162
**Apoptosis process**						
survivin	Increased	Poor prognosis	Dobrzycka, B., et al. (2015)^31^	survivin	SOC	92
Smac/DIABLO	Decreased	Poor prognosis	Dobrzycka, B., et al. (2015)^31^	Smac/DIABLO	SOC	92
**Others**						
miR-200c, miR-141	Increased	Good prognosis	Gao, Y.C., et al. (2015)^49^	miR-200C, miR-141	EOC	93
Platelet counts	Increased	Poor prognosis	Chen, Y., et al. (2015)^50^	Platelet counts	EOC	816
SFRA	Increased	Poor prognosis	Kurosaki, A., et al. (2016)^51^	SFRA	EOC	128
OPN	Increased	Poor prognosis	Zivny, J. H., et al. (2016)^52^	OPN	SOC	66
microRNA-125b (miR-125b)	Increased	Poor prognosis	Zuberi, M., et al. (2016)^53^	microRNA-125b (miR-125b)	EOC	70
miR-125b	Increased	Good prognosis	Zhu, T., et al. (2017)^54^	miR-125b	EOC	135
BGA	Expression	Good prognosis	Montavon Sartorius, C., et al. (2018)^55^	BGA	OC	282
RASSF1A rs1989839C > T SNP	Increased	Poor prognosis	He, W., et al. (2018)^56^	RASSF1A rs1989839C > T SNP	OC	1375
MACC1 and S100A4 transcripts	Increased	Poor prognosis	Link, T., et al. (2019)^57^	MACC1 and S100A4 transcripts	OC	79
sP (Hyp-Leu,Glu-Phe-Trp)	Decreased	Good prognosis	Lu, X., et al. (2019)^58^	sP (Hyp-Leu,Glu-Phe-Trp)	EOC	98

**Abbreviations:** miR: MicroRNA; NLR: the ratio of neutrophil count to lymphocyte count; AFR: albumin-to-fibrinogen ratio; PLR: platelet lymphocyte ratio; SNP: single Nucleotide Polymorphism; MSLN: Mesothelin; AAK: Aurora A kinase; Gal: Galectin; VEGF: vascular endothelial growth factor; sPD-L1: soluble PD - L1; OC: ovarian cancer; HGSOC: High grade serous ovarian cancer; EOC: epithelial ovarian cancer.

**Table 2:** Tissue-based immunohistochemistry biomarkers in ovarian cancer

	Expression or ratio	Potential clinical use	Example study			
			Study	Studied biomarkers	Subsite	Patients (n)
**EMT and metastasis**						
CTHRC1	Increased	Poor prognosis	Hou, M., et al. (2015)^59^	CTHRC1	EOC	88
ZEB2	Increased	Poor prognosis	Prislei, S., et al. (2015)^60^	ZEB2	EOC	143
CD44v6	Increased	Poor prognosis	Tjhay, F., et al. (2015)^61^	CD44v6	EOC	59
miR-506	Increased	Good prognosis	Sun, Y., et al. (2015)^62^	miR-506	EOC	204
FILIP1L	Increased	Good prognosis	Kwon, M., et al. (2016)^63^	FILIP1L	OC	369
Par3	Decreased	Good prognosis	Nakamura, H., et al. (2016)^64^	Par3	OC	50
MMP-14, CD44	Double expression	Poor prognosis	Vos, M. C., et al. (2016)^65^	MMP-14, CD44	OC	97
OTUB1	Expression	Poor prognosis	Wang, Y., et al. (2016)^66^	OTUB1	OC	200
ESRP1	Increased	Good prognosis	Chen, L., et al. (2017)^67^	ESRP1	EOC	109
MDM2	Increased	Good prognosis	Chen, Y., et al. (2017)^68^	MDM2	OC	104
CD24	Increased	Poor prognosis	Nakamura, K., et al. (2017)^69^	CD24	OC	174
CCNG1	Increased	Poor prognosis	Xu, Y., et al. (2019)^70^	CCNG1	HGSOC	266
DDR2	Increased	Poor prognosis	Ramalho, S., et al. (2019)^71^	DDR2	HGSOC	78
**Inflammation and immune response**						
CD8/Treg ratio	Increased	Good prognosis	Knutson, K. L., et al. (2015)^72^	CD8/Treg ratio	EOC	405
PD-1, PD-L1	Increased	Good prognosis	Darb-Esfahani, S., et al. (2016)^73^	PD-1, PD-L1	HGSOC	215
Tumour-infiltrating B cell and plasma cell	Increased	Poor prognosis	Lundgren, S., et al. (2016)^74^	Tumour-infiltrating B cell and plasma cell	EOC	154
TIL	Increased	Good prognosis	James, F. R., et al. (2017)^75^	TIL	EOC	707
T-bet+ TILs	Increased	Good prognosis	Xu, Y., et al. (2017)^76^	T-bet^+^ TILs	EOC	81
PD-L1	Increased	Poor prognosis	Zhu, J., et al. (2017)^77^	PD-L1	OCCC	138
Transcription factors WT1 and p53	Increased	Poor prognosis	Carter, J. H., et al. (2018)^78^	Transcription factors WT1 and p53	OC	96
SOCS-1	Increased	Poor prognosis	Nakagawa, S., et al. (2018)^79^	SOCS-1	OC	83
PD-L1	Increased	Good prognosis	Kim, K. H., et al. (2019)^80^	PD-L1	EOC	248
TIL	Increased	Good prognosis	Mauricio, P., et al. (2019)^81^	TIL	HGSOC	128
RCAS1-Ir	Increased	Poor prognosis	Szubert, S., et al. (2019)^82^	RCAS1-Ir	EOC	67
VISTA	Expression	Good prognosis	Zong, L., et al. (2020)^83^	VISTA	OC	146
Co-expression of CD8^+^ and granzyme B^+^	Increased	Good prognosis	Jäntti, T., et al. (2020)^84^	Co-expression of CD8^+^ and granzyme B^+^	HGSOC	67
**Antioxidant**						
Nrf2	Expression	Poor prognosis	Liew, P. L., et al. (2015)^85^	Nrf2	OC	108
SOD2	Increased	Poor prognosis	Amano, T., et al. (2019)^86^	SOD2	EAOC	61
**Angiogenesis**						
pIKK	Expression	Poor prognosis	Kinose, Y., et al. (2015)^87^	pIKK	OC	94
PDGFβR	Increased	Poor prognosis	Corvigno, S., et al. (2016)^88^	PDGFβR	SOC	186
VEGF-R1, VEGF-R2	Expression	Good prognosis	Skirnisdottir, I., et al. (2016)^89^	VEGF-R1, VEGF-R2	EOC	131
Nestin	Increased	Poor prognosis	Onisim, A., et al. (2016)^90^	Nestin	SOC	85
MIG-7	Increased	Poor prognosis	Huang, B., et al. (2016)^91^	MIG-7	EOC	121
PTEN	Expression	Good prognosis	Shen, W., et al. (2017)^92^	PTEN	OC	76
HIF-lα and VEGF	Expression	Poor prognosis	Shen, W., et al. (2017)^92^	HIF-lα and VEGF	OC	76
AEG-1	Increased	Poor prognosis	Yu, X., et al. (2018)^93^	AEG-1	EOC	170
VEGF, SEMA4D	Expression	Poor prognosis	Chen, Y., et al. (2018)^94^	VEGF, SEMA4D	EOC	124
TBC1D16	Increased	Good prognosis	Yang, Z., et al. (2018)^95^	TBC1D16	EOC	156
PGF	Increased	Poor prognosis	Meng, Q., et al. (2018)^96^	PGF	EOC	89
VEGF-A	Decreased	Poor prognosis	Sopo, M., et al. (2019)^97^	VEGF-A	OC	86
vasohibin-1, MACC1	Increased	Poor prognosis	Yu, L., et al. (2019)^98^	vasohibin-1, MACC1	SOC	124
Tie-2	Increased	Poor prognosis	Sopo, M., et al. (2020)^99^	Tie-2	HGSOC	86
**Cell proliferation**						
FASN	Increased	Poor prognosis	Cai, Y., et al. (2015)^100^	FASN	OC	60
CD73	Increased	Poor prognosis	Turcotte, M., et al. (2015)^101^	CD73	HGSOC	208
SPINK1	Increased	Poor prognosis	Mehner, C., et al. (2015)^102^	SPINK1	EOC	490
KCNN4, S100A14	Increased	Poor prognosis	Zhao, H., et al. (2016)^103^	KCNN4, S100A14	SOC	127
EGFR	Increased	Poor prognosis	Xu, L., et al. (2016)^104^	EGFR	EOC	67
Gab1	Increased	Poor prognosis	Hu, L. and R. Liu (2016)^105^	Gab1	EOC	124
IL-36α	Decreased	Poor prognosis	Chang, L., et al. (2017)^106^	IL-36α	EOC	96
DOT1L	Increased	Poor prognosis	Zhang, X., et al. (2017)^107^	DOT1L	OC	250
KRT5, KRT6	Increased	Poor prognosis	Ricciardelli, C., et al. (2017)^108^	KRT5, KRT6	SOC	117
hLSR	Increased	Poor prognosis	Hiramatsu, K., et al. (2018)^109^	hLSR	EOC	104
PAUF**,** TIR4	TLR4^high^ and PAUF^high^/TLR4^high^	Poor prognosis	Choi, C. H., et al. (2018)^110^	PAUF**,** TIR4	EOC	205
PCDH8	Decreased	Poor prognosis	Cao, Y., et al. (2018)^111^	PCDH8	OC	68
RIF1	Increased	Poor prognosis	Liu, Y. B., et al. (2018)^112^	RIF1	EOC	72
FGFR2	Increased	Poor prognosis	Li, M., et al. (2018)^113^	FGFR2	OC	426
FOXO1/PAX3	Increased	Poor prognosis	Han, G. H., et al. (2019)^114^	FOXO1 / PAX3	EOC	212
pStat3	Increased	Poor prognosis	Li, H., et al. (2020)^115^	pStat3	EOC	156
ATAD2	Increased	Poor prognosis	Liu, Q., et al. (2020)^116^	ATAD2	OC	60
**Cell migration**						
GRO-β	Increased	Poor prognosis	Ye, Q., et al. (2015)^117^	GRO-β	OC	136
B7-H6	Increased	Poor prognosis	Zhou, Y., et al. (2015)^118^	B7-H6	OC	110
OCT4, Notch1 and DLL4	Increased	Poor prognosis	Yu, L., et al. (2016)^119^	OCT4, Notch1 and DLL4	EOC	207
EphA8	Increased	Poor prognosis	Liu, X., et al. (2016)^120^	EphA8	OC	233
AGTR1	Increased	Poor prognosis	Zhang, Q., et al. (2019)^121^	AGTR1	EOC	902
**Cell invasion**						
CK2α	Increased	Poor prognosis	Ma, Z., et al. (2017)^122^	CK2α	EOC	117
CEP55	Increased	Poor prognosis	Zhang, W., et al (2017)^123^	CEP55	EOC	213
ANXA1	Increased	Good prognosis	Manai, M., et al. (2020)^124^	ANXA1	EOC	156
**Cell proliferation and migration**						
MAP3K8	Increased	Poor prognosis	Gruosso, T., et al. (2015)^125^	MAP3K8	HGSOC	139
IL-33/ST2 axis	Increased	Poor prognosis	Tong, X., et al. (2016)^126^	IL-33/ST2 axis	EOC	152
CDCP1, ADAM12	Decreased	Good prognosis	Vlad, C., et al. (2016)^127^	CDCP1, ADAM12	SOC	102
FGFRL1	Increased	Poor prognosis	Tai, H., et al. (2018)^128^	FGFRL1	OC	90
HSDL2	Increased	Poor prognosis	Sun, Q., et al. (2018)^129^	HSDL2	OC	74
DUSP2	Decreased	Poor prognosis	Liu, W., et al. (2019)^130^	DUSP2	HGSOC	127
Kallistatin (KAL)	Decreased	Poor prognosis	Wu, H., et al. (2019)^131^	Kallistatin (KAL)	HGSOC	312
YTHDF1-EIF3C axis	Increased	Poor prognosis	Liu, T., et al. (2020)^132^	YTHDF1-EIF3C axis	OC	134
**Cell proliferation and invasion**						
IL-6R	Increased	Good prognosis	Isobe, A., et al. (2015)^133^	IL-6R	OC	94
Usp7, MARCH7	Increased	Poor prognosis	Zhang, L., et al. (2016)^134^	Usp7, MARCH7	EOC	121
PPA1	Increased	Poor prognosis	Li, H., et al. (2017)^135^	PPA1	SOC	139
PATZ1	Increased	Good prognosis	Zhao, C., et al. (2018)^136^	PATZ1	SOC	208
**Cell migration and invasion**						
ARMC8	Increased	Poor prognosis	Jiang, G., et al.(2015)^137^	ARMC8	OC	247
galectin-1	Increased	Poor prognosis	Chen, L., et al. (2015)^138^	galectin-1	EOC	110
MAGE-A9	Increased	Poor prognosis	Xu, Y., et al. (2015)^139^	MAGE-A9	EOC	128
TROP2	Increased	Poor prognosis	Xu, N., et al. (2016)^140^	TROP2	EOC	128
GALNT6	Increased	Poor prognosis	Lin, T. C., et al. (2017)^141^	GALNT6	EOC	78
Galectin-1	Increased	Poor prognosis	Schulz, H., et al. (2017)^142^	Galectin-1	OC	156
Galectin-3	Increased	Poor prognosis	Schulz, H., et al. (2017)^142^	Galectin-3	OC	156
Galectin-7	Increased	Good prognosis	Schulz, H., et al. (2017)^142^	Galectin-7	OC	156
REDD1	Increased	Poor prognosis	Chang, B., et al. (2018)^143^	REDD1	OC	229
RacGAP1	Decreased	Good prognosis	Wang, C., et al. (2018)^144^	RacGAP1	EOC	117
PAI-1, PAI-RBP1	Increased	Poor prognosis	Koensgen, D., et al. (2018)^145^	PAI-1, PAI-RBP1	OC	156
PRDX-1	Increased	Poor prognosis	Sienko, J., et al. (2019)^146^	PRDX-1	OC	55
KAI1	Decreased	Poor prognosis	Yu, L., et al. (2019)^98^	KAI1	SOC	124
CAV1, ATG4C	Increased	Poor prognosis	Zeng, Y., et al. (2020)^147^	CAV1, ATG4C	EOC	95
**Cell proliferation, migration and invasion**						
CH13L1, FKBP4	Increased	Poor prognosis	Lawrenson, K., et al. (2015)^148^	CH13L1, FKBP4	EOC	200
REG4	Increased	Poor prognosis	Chen, S., et al. (2015)^149^	REG4	EOC	337
Spry2	Decreased	Poor prognosis	Masoumi-Moghaddam, S., et al. (2015)^150^	Spry2	OC	99
SWI/SNF subunits	Decreased	Poor prognosis	Abou-Taleb, H., et al. (2016)^151^	SWI/SNF subunits	EOC	152
KIF2A	Decreased	Poor prognosis	Wang, D., et al. (2016)^152^	KIF2A	EOC	111
Salusin-β	Increased	Poor prognosis	Zhang,Q.,et al.(2017)^153^	Salusin-β	OC	57
P38α, ATF2	Increased	Poor prognosis	Song,W.J.,et al.(2017)^154^	P38α, ATF2	OSC	120
nERβ5	Increased	Poor prognosis	Chan, K. K. L., et al. (2017)^155^	nERβ5	OC	106
SENP3/SMT3IP1	Increased	Poor prognosis	Cheng, J., et al. (2017)^156^	SENP3/SMT3IP1	EOC	124
BCL6, Lewis y	Increased	Poor prognosis	Zhu, L., et al. (2017)^157^	BCL6, Lewis y	OC	103
CXCL11, HMGA2	Increased	Poor prognosis	Jin, C., et al. (2018)^158^	CXCL11, HMGA2	HGSOC	110
HS3ST2	Decreased	Poor prognosis	Huang, R.L., et al. (2018)^159^	HS3ST2	EOC	115
KIF2A	Increased	Poor prognosis	Sheng, N., et al. (2018)^160^	KIF2A	OC	108
TRIM59	Increased	Good prognosis	Wang, Y., et al. (2018)^161^	TRIM59	OC	192
S100A10	Increased	Poor prognosis	Wang, L., et al. (2019)^162^	S100A10	OC	138
PYGB	Increased	Poor prognosis	Zhou, Y., et al. (2019)^163^	PYGB	OC	94
**Glycosylation disorder of protein**						
GalNAs T6, T14	Increased	Poor prognosis	Sheta, R., et al. (2017)^164^	GalNAs T6, T14	HGSOC	131
**Mitotic process**						
TOPK	Increased	Poor prognosis	Ikeda, Y., et al. (2016)^165^	TOPK	EOC	163
HER2, AURKA	Increased	Poor prognosis	Li, M.J., et al. (2017)^166^	HER2, AURKA	OCCC	60
KIF14	Increased	Poor prognosis	Qiu, H. L., et al. (2017)^167^	KIF14	EOC	170
**Apoptosis process**						
PDCD5	Decreased	Poor prognosis	Gao, L., et al. (2015)^168^	PDCD5	OC	127
MDM2	Increased	Poor prognosis	Makii, C., et al. (2016)^169^	MDM2	OCCC	75
DNA-PKcs, Akt3, p53	Increased	Poor prognosis	Shin, K., et al. (2016)^170^	DNA-PKcs, Akt3, p53	SOC	132
Gal-1, Gal-8, Gal-9p	Increased	Poor prognosis	Labrie, M., et al. (2017)^171^	Gal-1, Gal-8, Gal-9p	HGSOC	209
**Cell survival (telomerase activity)**						
Phosphorylated Akt, hTERT	Increased	Poor prognosis	Lee, Y. K., et al. (2015)^172^	phosphorylated Akt, hTERT	EOC	92
**Chemotherapeutic sensitivity**						
JARID1B	Increased	Poor prognosis	Wang, L., et al. (2015)^173^	JARID1B	EOC	120
ALDH1	Increased	Good prognosis	Ayub, T. H., et al. (2015)^174^	ALDH1	EOC	55
PRP4K	Increased	Good prognosis	Corkery, D. P., et al. (2015)^175^	PRP4K	OC	199
HtrA2	Decreased	Poor prognosis	Miyamoto, M., et al. (2015)^176^	HtrA2	HGSOC	142
PTEN	Increased	Good prognosis	Wang, L., et al. (2015)^177^	PTEN	EOC	161
NF-κBp65	Increased	Poor prognosis	Wang, L., et al. (2015)^177^	NF-κBp65	EOC	161
eIF3a	Increased	Good prognosis	Zhang, Y., et al. (2015)^178^	eIF3a	OC	126
GTF2H5	Decreased	Good prognosis	Gayarre, J., et al. (2016)^179^	GTF2H5	HGSOC	117
POSTN	Increased	Poor prognosis	Sung, P. L., et al. (2016)^180^	POSTN	EOC	308
SOX10	Increased	Poor prognosis	Know, A.Y., et al. (2016)^181^	SOX10	EOC	203
GOLPH3L	Increased	Poor prognosis	He, S., et al. (2017)^182^	GOLPH3L	OC	177
LC3A	Increased	Poor prognosis	Miyamoto, M., et al. (2017)^183^	LC3A	OCCC	117
Stonin 2 (STON2)	Increased	Poor prognosis	Sun, X., et al. (2017)^184^	Stonin 2 (STON2)	EOC	89
GATA3	Increased	Poor prognosis	Chen, H. J., et al. (2018)^185^	GATA3	OC	196
EpCAM	Increased	Poor prognosis	Zhang, X., et al. (2018)^186^	EpCAM	EOC	109
UBC13	Decreased	Poor prognosis	Zhang, X., et al. (2018)^187^	UBC13	OC	71
14-3-3ζ	Increased	Poor prognosis	Kim, H. J., et al. (2018)^188^	14-3-3ζ	OC	88
KCNN3	Increased	Poor prognosis	Liu, X., et al. (2018)^189^	KCNN3	OC	57
HELQ	Increased	Poor prognosis	Long, J., et al. (2018)^190^	HELQ	EOC	87
P15 PAF (KIAA0101)	Increased	Poor prognosis	Jin, C., et al. (2018)^191^	P15 PAF (KIAA0101)	HGSOC	118
UTP23	Decreased	Poor prognosis	Fu, Z., et al. (2019)^192^	UTP23	OC	133
ABCB9	Decreased	Poor prognosis	Hou, L., et al. (2019)^193^	ABCB9	OC	308
PBK	Increased	Poor prognosis	Ma, H., et al. (2019)^194^	PBK	HGSOC	234
Sorcin	Decreased	Good prognosis	Zhang, S., et al. (2019)^195^	Sorcin	OC	60
PRC1	Increased	Poor prognosis	Bu, H., et al. (2020)^196^	PRC1	HGSOC	210
NCALD	Decreased	Poor prognosis	Feng, L. Y. and L. Li (2020)^197^	NCALD	EOC	239
**Cell cycle regulation**						
CAP1	Increased	Poor prognosis	Hua, M., et al. (2015)^198^	CAP1	EOC	119
CCNE1	Increased	Poor prognosis	Ayhan, A., et al. (2017)^199^	CCNE1	OCCC	120
NUCKS	Increased	Poor prognosis	Shi, C., et al. (2017)^200^	NUCKS	OC	121
TK1	Increased	Poor prognosis	Wang, J., et al. (2017)^201^	TK1	SOC	109
**Differentiation of cancer-associated fibroblasts (CAFs)**						
MARCKS	Increased	Poor prognosis	Doghri, R., et al. (2017)^202^	MARCKS	EOC	118
**Immunosuppression**						
VEGF	Increased	Poor prognosis	Horikawa, N., et al. (2017)^203^	VEGF	HGSOC	56
**Metabolic reprogramming**						
TBC1D8	Increased	Poor prognosis	Chen, M., et al. (2019)^204^	TBC1D8	OC	141
**Fatty acid metabolism**						
PAX2	Increased	Poor prognosis	Feng, Y., et al. (2020)^205^	PAX2	EOC	152
**Defective DNA repair**						
WRAP53β	Decreased	Poor prognosis	Hedström, E., et al. (2015)^206^	WRAP53β	EOC	151
pH2AX	Increased	Poor prognosis	Mei, L., et al. (2015)^207^	pH2AX	EOC	87
**Others**						
SLP-2	Increased	Poor prognosis	Sun, F., et al. (2015)^208^	SLP-2	EOC	140
CD44v8-10	Expression	Good prognosis	Sosulski, A., et al. (2016)^209^	CD44v8-10	SOC	210
P53	Increased	Poor prognosis	Zuo, J., et al. (2016)^210^	P53	SOC	183
Highly sulfated CS	Increased	Poor prognosis	Van der steen, S.C., et al. (2016)^211^	Highly sulfated CS	EOC	255
Adiponectin receptor-1 (AdipoR1)	Increased	Good prognosis	Li, X., et al. (2017)^212^	Adiponectin receptor-1 (AdipoR1)	EOC	73
TP53	Increased	Poor prognosis	Rzepecka, I. K., et al. (2017)^213^	TP53	HGSOC	159
SMAD3	Increased	Poor prognosis	Sakr, S., et al. (2017)^214^	SMAD3	GCT	88
ALDH5A1	Increased	Good prognosis	Tian, X., et al. (2017)^215^	ALDH5A1	OC	192
GR	Increased	Poor prognosis	Veneris, J. T., et al. (2017)^216^	GR	EOC	341
LAMP3	Increased	Poor prognosis	Wang, D., et al. (2017)^217^	LAMP3	EOC	135
HBXIP	Increased	Poor prognosis	Wang, Y., et al. (2017)^218^	HBXIP	OC	120
HSF1 pSer326	Expression	Poor prognosis	Yasuda, K., et al. (2017)^219^	HSF1 pSer326	EOC	122
COX-1, COX-2	Increased	Poor prognosis	Beeghly-Fadiel, A., et al. (2018)^220^	COX-1, COX-2	EOC	190
GPR30	Expression	Poor prognosis	Zhu, C. X., et al. (2018)^221^	GPR30	EOC	110
HJURP	Increased	Poor prognosis	Li, L., et al. (2018)^222^	HJURP	HGSOC	98
Galectins-8	Increased	Good prognosis	Schulz, H., et al. (2018)^223^	Galectins-8	OC	156
HER3	Expression	Poor prognosis	Chung, Y. W., et al. (2019)^224^	HER3	EOC	105
ANXA8	Increased	Poor prognosis	Gou, R., et al. (2019)^225^	ANXA8	OC	122
USP10/p14ARF	Decreased	Poor prognosis	Han, G. H., et al. (2019)^226^	USP10/p14ARF	EOC	212
PKP3	Increased	Poor prognosis	Qian, H., et al. (2019)^227^	PKP3	OC	157
PDGFR-β	Expression	Good prognosis	Szubert, S., et al. (2019)^228^	PDGFR-β	EOC	52
CN	Increased	Poor prognosis	Xin, B., et al. (2019)^229^	CN	OC	50
TSLP	Increased	Poor prognosis	Xu, L., et al. (2019)^230^	TSLP	EOC	144
BUB1B, KIF11 and KIF20A	Increased	Poor prognosis	Zhang, L., et al. (2019)^231^	BUB1B, KIF11 and KIF20A	OC	50
VDR	Increased	Poor prognosis	Czogalla, B., et al. (2020)^232^	VDR	EOC	156

**Abbreviations:** TIL: tumor infiltrates lymphocytes; Gal: Galectin; OC: ovarian cancer; HGSOC: High grade serous ovarian cancer; EOC: epithelial ovarian cancer.

**Table 3:** Tissue-based DNA biomarkers in ovarian cancer

	Expression or ratio	Potential clinical use	Example study				
			Study	Studied biomarkers	Method	Subsite	Patients (n)
**Methylation**							
MYLK3 Methylation	Increased	Good prognosis	Phelps, D.L., et al. (2017)^233^	MYLK3 Methylation	Pyrosequencing	SOC	803
HNF1B	Expression	Poor prognosis	Bubancova, I., et al. (2017)^234^	HNF1B	NGS, HRM, MS-PCR	OC	64
GATA4	Expression	Good prognosis	Bubancova, I., et al. (2017)^234^	GATA4	NGS, HRM, MS-PCR	OC	64
HS3ST2	Increased	Poor prognosis	Huang, R.L., et al. (2018)^159^	HS3ST2	TMA	EOC	115
ZNF671	Increased	Early relapse	Mase, S., et al. (2019)^235^	ZNF671	Pyrosequencing	HGSOC	78
**Structural changes of nuclear chromatin**							
Chromatin entropy nuclei	Increased	Poor prognosis	Nielsen, B. et al. (2018)^236^	Chromatin entropy nuclei	Nuclear Texture analysis	OC	246
**Mutation status**							
BRCA1/2 wild type	Expression	Poor prognosis	Eoh, K. J., et al. (2017)^237^	BRCA1/2 wild type	Direct sequencing	EOC	116
BRCA1/2	Expression	Good prognosis	Kim, S. I., et al. (2019)^238^	BRCA1/2	Sanger sequencing	HGSOC	128
**Cell proliferation and apoptosis**							
ecDNA	Increased	Poor prognosis	Kalavska, K., et al. (2018)^239^	ecDNA	RT-PCR	OC	67
**Gene polymorphism**							
The AT genotype of rs189897	Expression	Poor prognosis	Liu, J., et al. (2019)^240^	The AT genotype of rs189897	Mass ARRAY	EOC	200
rs12921862 C/C	Expression	Good prognosis	Zhang, Y., et al. (2019)^241^	rs12921862 C/C	PCR-RFLP	EOC	165

**Abbreviations:** TMA: tissue microarrays; NGS: Next Generation Sequencing; MS-PCR: Methylation-Specific PCR; RT-PCR: real time polymerase chain reaction; PCR-RFLP: polymerase chain reaction-restriction fragment length polymorphism.

**Table 4:** Tissue-based RNA biomarkers in ovarian cancer

	Expression or ratio	Potential clinical use	Example study				
			Study	Studied biomarkers	Method	Subsite	Patients (n)
**Cell proliferation**							
microRNA(miR)-498	Decreased	Poor prognosis	Cong, J., et al. (2015)^242^	microRNA(miR)-498	qRT-PCR	OC	175
miR-193b	Decreased	Poor prognosis	Li, H., et al. (2015)^243^	miR-193b	qRT-PCR	OC	116
miR-572	Decreased	Good prognosis	Zhang, X., et al. (2015)^244^	miR-572	qRT-PCR	OC	108
C7	Decreased	Poor prognosis	Ying, L., et al. (2016)^245^	C7	qRT-PCR	OC	156
HER2, STAT3	Increased	Poor prognosis	Shang, A. Q., et al. (2017)^246^	HER2, STAT3	qRT-PCR	OC	136
SOCS3	Decreased	Poor prognosis	Shang, A. Q., et al. (2017)^246^	SOCS3	qRT-PCR	OC	136
lncRNA RAD51-AS1	Increased	Poor prognosis	Zhang, X., et al. (2017)^247^	lncRNA RAD51-AS1	qRT-PCR	EOC	163
lncRNA LINC 00152	Increased	Poor prognosis	Chen, P., et al. (2018)^248^	lncRNA LINC 00152	qRT-PCR	OC	82
miR-1294	Increased	Good prognosis	Guo, T. Y., et al. (2018)^249^	miR-1294	qRT-PCR	EOC	76
lncRNA TUG1	Increased	Poor prognosis	Li, T. H., et al. (2018)^250^	lncRNA TUG1	qRT-PCR	EOC	96
microRNA-424-5p (miR-424-5p)	Increased	Good prognosis	Liu, J., et al. (2018)^251^	microRNA-424-5p (miR-424-5p)	qRT-PCR	EOC	83
**Cell migration**							
lncRNA LINC00092	Increased	Poor prognosis	Zhao, L., et al. (2017)^252^	lncRNA LINC00092	qRT-PCR	SOC	58
lncRNA PTPRG-AS1	Increased	Poor prognosis	Ren, X. Y., et al. (2020)^253^	lncRNA PTPRG-AS1	qRT-PCR	EOC	184
**Cell invasion**							
lncRNA NEAT1	Increased	Poor prognosis	Chen, Z. J., et al. (2016)^254^	lncRNA NEAT1	qRT-PCR	OC	149
ASAP1-IT1	Increased	Good prognosis	Fu, Y., et al. (2016)^255^	ASAP1-IT1	qRT-PCR	EOC	266
**Cell proliferation and migration**							
miR-145	Decreased	Poor prognosis	Kim,T.H.,et al.(2015)^256^	miR-145	qRT-PCR	HGSOC	74
microRNA-196a	Increased	Poor prognosis	Fan, Y., et al. (2015)^257^	microRNA-196a	qRT-PCR	EOC	156
miR-552	Increased	Poor prognosis	Zhao, W., et al. (2019)^258^	miR-552	qRT-PCR	OC	110
**Cell proliferation and invasion**							
lncRNA AB073614	Increased	Poor prognosis	Cheng, Z., et al. (2015)^259^	lncRNA AB073614	qRT-PCR	OC	75
TBL1XR1	Increased	Poor prognosis	Ma, M. and N. Yu (2017)^260^	TBL1XR1	qRT-PCR	SOC	116
lncRNA MNX1-AS1	Increased	Poor prognosis	Li, A. H. and H. H. Zhang (2017)^261^	lncRNA MNX1-AS1	qRT-PCR	EOC	177
lncRNA NEAT1	Increased	Poor prognosis	Yong, W., et al. (2018)^262^	lncRNA NEAT1	qRT-PCR	HGSOC	75
miR-532-5p	Decreased	Poor prognosis	Wei, H., et al. (2018)^263^	miR-532-5p	qRT-PCR	EOC	145
**Cell migration and invasion**							
ANRIL	Increased	Poor prognosis	Qiu,J.J.,et al.(2015)^264^	ANRIL	qRT-PCR	SOC	68
lncRNA CCAT1	Increased	Poor prognosis	Cao,Y.,et al.(2017)^265^	lncRNA CCAT1	qRT-PCR	EOC	72
miR-208a-5p	Increased	Good prognosis	Mei, J., et al. (2019)^266^	miR-208a-5p	qRT-PCR	OC	61
STAT2	Increased	Poor prognosis	Chen, X., et al. (2020)^267^	STAT2	RT-PCR	OC	62
lncRNA miR503HG	Decreased	Poor prognosis	Zhu, D., et al. (2020)^268^	lncRNA miR503HG	qRT-PCR	OC	61
**Cell proliferation, migration and invasion**							
lncRNA CCAT2	Increased	Poor prognosis	Huang,S.,et al.(2016)^269^	lncRNA CCAT2	qRT-PCR	OC	109
GOLPH3	Increased	Poor prognosis	Sun, J., et al. (2017)^270^	GOLPH3	qRT-PCR	EOC	73
lncRNA HOXA11as	Increased	Poor prognosis	Yim, G. W., et al. (2017)^271^	lncRNA HOXA11as	qRT-PCR	SOC	129
miR-520h	Increased	Poor prognosis	Zhang, J., et al. (2018)^272^	miR-520h	qRT-PCR	EOC	116
lncRNA SNHG16	Increased	Poor prognosis	Yang, X. S., et al. (2018)^273^	lncRNA SNHG16	qRT-PCR	OC	103
lncRNA EBIC	Increased	Poor prognosis	Xu, Q. F., et al. (2018)^274^	lncRNA EBIC	qRT-PCR	OC	126
lncRNA MALAT1	Increased	Poor prognosis	Guo, C., et al. (2018)^275^	lncRNA MALAT1	qRT-PCR	OC	60
lncRNA RP11-552M11.4	Increased	Poor prognosis	Huang, K., et al. (2018)^276^	lncRNA RP11-552M11.4	qRT-PCR	EOC	67
lncRNA OTUB1-isoform2	Increased	Poor prognosis	Wang, S., et al. (2018)^277^	lncRNA OTUB1-isoform2	qRT-PCR	OC	114
HYOU1	Increased	Poor prognosis	Li, X., et al. (2019)^278^	HYOU1	qRT-PCR	EOC	127
miR-203a-3p	Increased	Good prognosis	Liu, H. Y., et al. (2019)^279^	miR-203a-3p	qRT-PCR	OC	152
LINC00339	Increased	Poor prognosis	Pan, L., et al. (2019)^280^	LINC00339	qRT-PCR	OC	75
lncRNA SNHG20	Increased	Poor prognosis	Wang, D., et al. (2019)^281^	lncRNA SNHG20	RT-PCR	EOC	60
miR-149	Increased	Good prognosis	Zhao, L. W., et al. (2020)^282^	miR-149	qRT-PCR	OC	72
**Chemotherapeutic sensitivity**							
microRNA-506 (miR-506)	Increased	Good prognosis	Liu, G., et al. (2015)^283^	microRNA-506 (miR-506)	qRT-PCR	EOC	598
CHI3L1	Increased	Poor prognosis	Chiang, Y. C., et al. (2015)^284^	CHI3L1	qRT-PCR	EOC	180
IMP3	Increased	Poor prognosis	Hsu, K. F., et al. (2015)^285^	IMP3	qRT-PCR	EOC	140
Lin28B	Increased	Poor prognosis	Hsu, K. F., et al. (2015)^285^	Lin28B	qRT-PCR	EOC	140
Tribbles 2 (TRIB2)	Decreased	Poor prognosis	Kritsch, D., et al. (2017)^286^	Tribbles 2 (TRIB2)	qRT-PCR	EOC	149
let-7e	Decreased	Poor prognosis	Xiao, M., et al. (2017)^287^	let-7e	qRT-PCR	EOC	84
MAL	Increased	Poor prognosis	Zanotti, L., et al. (2017)^288^	MAL	qRT-PCR	HGSOC	74
miR-98-5p	Increased	Good prognosis	Wang, Y., et al. (2018)^289^	miR-98-5p	qRT-PCR	EOC	97
miR-1180	Increased	Poor prognosis	Gu, Z. W., et al. (2019)^290^	miR-1180	qRT-PCR	OC	59
lncRNA GAS5	Increased	Good prognosis	Long, X., et al. (2019)^291^	lncRNA GAS5	qRT-PCR	EOC	53
**Immune response**							
APOBEC3G	Increased	Good prognosis	Leonard, B., et al. (2016)^292^	APOBEC3G	qRT-PCR	HGSOC	354
lncRNA MIR155HG	Increased	Good prognosis	Colvin, E. K., et al. (2020)^293^	lncRNA MIR155HG	qRT-PCR	HGSOC	67
**Chromosome structure and function**							
SMYD3 genetic polymorphisms	Expression	Poor prognosis	Liu, T. T., et al. (2016)^294^	SMYD3 genetic polymorphisms	qRT-PCR	OC	154
**Apoptosis process**							
CPS1-IT1	Increased	Good prognosis	Wang, Y. S., et al. (2017)^295^	CPS1-IT1	qRT-PCR	EOC	91
**Others**							
CRNDE	Increased	Poor prognosis	Szafron, L. M., et al. (2015)^296^	CRNDE	qRT-PCR	OC	135
GADD45A (1506T> C)	Increased	Poor prognosis	Yuan, C., et al. (2015)^297^	GADD45A (1506T> C)	qRT-PCR	OC	258
miR-510, miR-129-3P	Decreased	Poor prognosis	Zhang,X.,et al.(2015)^298^	miR-510, miR-129-3P	RT-qPCR,ISH	EOC	78
FAM215A	Increased	Good prognosis	Fu, Y., et al. (2016)^255^	FAM215A	qRT-PCR	EOC	266
LIN-28B/let-7a/IGF-II axis	LIN-28B^low^let-7a^low^ or LIN-28B^low^let-7a^high^IGF-II^low^	Good prognosis	Lu, L., et al. (2016)^299^	LIN-28B/let-7a/IGF-II axis	qRT-PCR	EOC	211
miR-200b, miR-1274A (tRNA Lys5) and miR-141	Decreased	Good prognosis	Halvorsen, A. R., et al. (2017)^300^	miR-200b, miR-1274A (tRNA Lys5) and miR-141	qRT-PCR	OC	207
miR-595	Decreased	Poor prognosis	Zhou, Q. H., et al. (2017)^301^	miR-595	qRT-PCR	EOC	166
KLK11, KLK15	Increased	Good prognosis	Geng,X.,et al.(2017)^302^	KLK11, KLK15	RT-PCR	HGSOC	139
lncRNA LINC01088	Decreased	Poor prognosis	Ai, H., et al. (2018)^303^	lncRNA LINC01088	qRT-PCR	EOC	184
lncRNA HMMR-AS1	Increased	Poor prognosis	Chu, Z. P., et al. (2018)^304^	lncRNA HMMR-AS1	qRT-PCR	EOC	152
circ LARP4	Decreased	Poor prognosis	Zou, T., et al. (2018)^305^	circ LARP4	qRT-PCR	OC	78
circ HIPK3	Increased	Poor prognosis	Liu, N., et al. (2018)^306^	circ HIPK3	qRT-PCR	EOC	69
lncRNA DGCR5	Decreased	Poor prognosis	Chen, H., et al. (2019)^307^	lncRNA DGCR5	qRT-PCR	OC	66
FANCD2	Increased	Poor prognosis	Moes-Sosnowska, J., et al. (2019)^308^	FANCD2	qRT-PCR	OC	99
AK7	Decreased	Poor prognosis	Zhang, X. Y., et al. (2021)^309^	AK7	RNAseq	OC	308

**Abbreviations:** lnc: Long non-coding RNA; circ: circular; qRT-PCR: quantitative real time polymerase chain reaction; RT-PCR: real time polymerase chain reaction; IHC: Immunohistochemistry; ISH, *in situ* hybridization.
