# Supplementary material for: Research Progress in Prognostic Factors and Biomarkers of Ovarian Cancer
Source: J Cancer. 2021 May 13;12(13):3976–96. doi: 10.7150/jca.47695 (PMC8176232; doi:10.7150/jca.47695)
Supplement: Supplementary file 1 — Supplementary materials. [file jcav12p3976s1.pdf]

Supplementary appendix

1. Search strategy

(((ovarian neoplasms [Title/Abstract]) OR (ovarian cancer [Title/Abstract])) OR (ovarian malignant tumor [Title/Abstract])) AND (((((prognosis [Title/Abstract]) OR (prognoses [Title/Abstract])) OR (prognostic [Title/Abstract])) OR (prognostic factors [Title/Abstract])) OR (prognostic factor [Title/Abstract])) Sort by: Best Match Filters: Free full text; published between February 1, 2015 and February 1, 2021; Humans

2. Selection criteria

**Inclusion criteria**	Ovarian cancer patients who have received surgery and/or radio(chemo)therapy as part of their treatment
	The prognostic value of novel prognostic markers was reported in the studies
	All the studies were published between February 1, 2015 and February 1, 2021
**Exclusion criteria**	The number of patients was less than 50 in these studies
	Prognostic factors of other diseases unrelated to ovarian cancer
	Prognostic factors were studied only in cell lines and/or animal models
	Systematic reviews and critical articles
	Studies had no positive results
	Studies not associated with clinical outcomes
